# Detection of DNA of filariae closely related to *Mansonella perstans* in faecal samples from wild non-human primates from Cameroon and Gabon

**DOI:** 10.1186/s13071-020-04184-1

**Published:** 2020-06-16

**Authors:** Colette Marie Gaillard, Sebastien David Pion, Hadjira Hamou, Constant Sirima, Charlotte Bizet, Thomas Lemarcis, Jules Rodrigues, Amandine Esteban, Martine Peeters, Eitel Mpoudi Ngole, Illich Mombo, Florian Liégeois, Coralie Martin, Michel Boussinesq, Sabrina Locatelli

**Affiliations:** 1 IRD UMI 233-INSERM U1175, University of Montpellier, Montpellier, France; 2 Unité Molécules de Communication et Adaptation des Microorganismes (MCAM UMR7245), Muséum national d’Histoire naturelle, CNRS, Paris, France; 3 Projet Prévention du Sida au Cameroun (PRESICA) and Virology Laboratory IMPM/IRD, Yaoundé, Cameroon; 4 Centre International de Recherches Médicales, BP 769 Franceville, Gabon; 5 Laboratoire Maladies Infectieuses et Vecteurs: Ecologie, Génétique, Evolution, Contrôle, UMR 224 IRD/CNRS/UM1, 34394 Montpellier, France

**Keywords:** *Mansonella* spp., Anthropoids, Phylogeny, Africa, Faeces, Gastro-intestinal worms, Zoonosis

## Abstract

**Background:**

The Onchocercidae is a family of filarial nematodes with several species of medical or veterinary importance. Microfilariae are found in the blood and/or the dermis and are usually diagnosed in humans by microscopy examination of a blood sample or skin biopsy. The main objectives of this study were to evaluate whether filariae DNA can be detected in faecal samples of wild non-human primates (NHPs), whether the detected parasites were closely related to those infecting humans and whether filarial DNA detection in faeces is associated with co-infections with nematodes (*Oesophagostumum* sp. and *Necato*r sp.) known to cause blood loss while feeding on the host intestinal mucosa.

**Methods:**

A total of 315 faecal samples from 6 species of NHPs from Cameroon and Gabon were analysed. PCRs targeted DNA fragments of *cox*1 and *12S* rDNA genes, to detect the presence of filariae, and the internal transcribed spacer 2 (ITS2), to detect the presence of *Oesophagostomum* sp. and *Necator* sp. infections.

**Results:**

Among the 315 samples analysed, 121 produced sequences with > 90% homology with Onchocercidae reference sequences. However, 63% of the *12S* rDNA and 78% of the *cox*1 gene sequences were exploitable for phylogenetic analyses and the amplification of the *12S* rDNA gene showed less discriminating power than the amplification of the *cox*1 fragment. Phylogenetic analyses showed that the *cox*1 sequences obtained from five chimpanzee DNA faecal samples from Gabon and two from Cameroon cluster together with *Mansonella perstans* with high bootstrap support. Most of the remaining sequences clustered together within the genus *Mansonella*, but the species could not be resolved. Among the NHP species investigated, a significant association between filarial DNA detection and *Oesophagostomum* sp. and *Necator* sp. infection was observed only in gorillas.

**Conclusions:**

To our knowledge, this is the first study reporting DNA from *Mansonella* spp. in faecal samples. Our results raise questions about the diversity and abundance of these parasites in wildlife, their role as sylvatic reservoirs and their potential for zoonotic transmission. Future studies should focus on detecting variants circulating in both human and NHPs, and improve the molecular information to resolve or support taxonomy classification based on morphological descriptions.
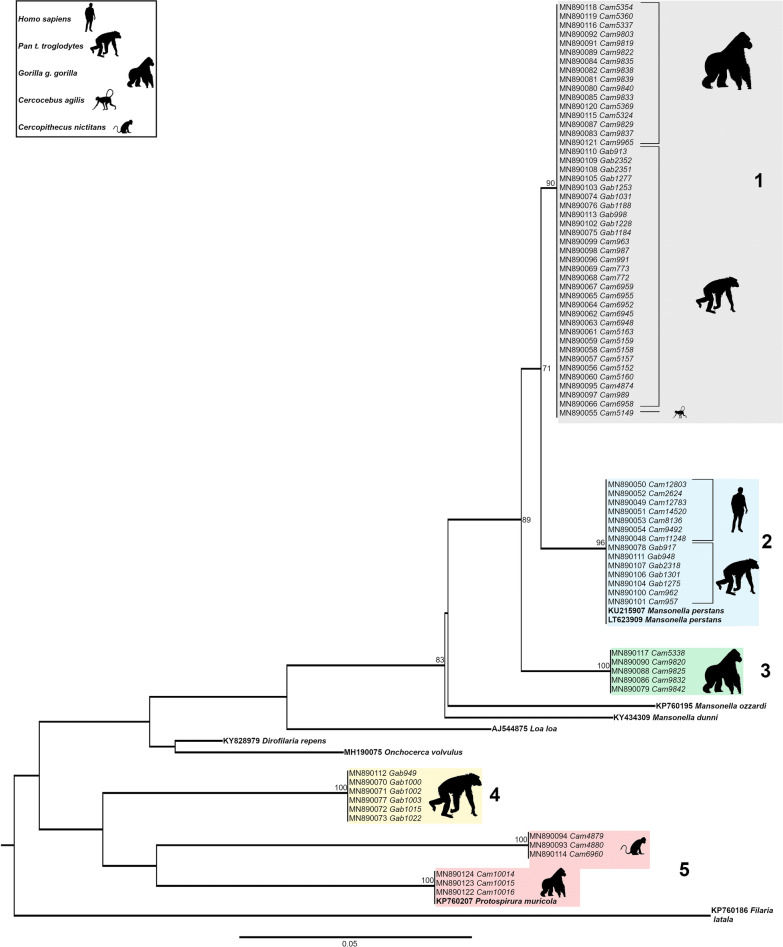

## Background

Onchocercidae nematodes form a family of parasitic worms comprising eight subfamilies and 88 genera [1, 2]. Species belonging to genera such as *Loa*, *Onchocerca*, *Wuchereria*, *Brugia* and *Mansonella* are responsible for human or animal diseases such as loiasis, onchocerciasis or lymphatic filariasis. Some of these species are found in both humans and non-human primates (NHPs) [2]. The diagnosis of filariasis is typically based on the detection of the adult stage of the parasite or, more often, of the larval stages produced by adult female worms, called microfilariae (mf). Several diagnostic methods are available to detect these parasites in humans. If one considers the main species parasitizing humans, mf are detected by light microscopy examination of a blood sample (*Loa loa*, *Wuchereria bancrofti*, *Brugia malayi* and *Mansonella perstans*), or of a skin biopsy (*Onchocerca volvulus* and *M. streptocerca*), or both (*M. ozzardi*) [3]. In addition to blood and dermis, mf can sometimes be found in the urine. Besides the microscopical identification of the parasites, various other techniques detecting antigens, antibodies or parasitic DNA are also available and used either for the individual diagnosis or to assess the infection endemicity levels in communities.

Molecular diagnostic methods, based on the amplification of parasite DNA by polymerase chain reaction (PCR) methods [4], or by loop-mediated isothermal amplification (LAMP) [5–7] have the advantages of being more sensitive in detecting parasites than the usual microscopy methods, especially in case of low mf densities, as well as increased certainty in the identification to the species or even strain level [2, 8].

In the early 2010s, it was observed that *Plasmodium* DNA could be found in NHP faeces [9], despite the fact that these are blood-borne parasites. Similar results reporting detectable amounts of *P. falciparum* DNA in the faeces of infected humans have been recently reported [10, 11]. However, the mechanisms underlying how *Plasmodium* DNA ends up in the faeces remain unclear. These observations led us to the question of whether PCR techniques could also effectively detect filarial DNA in faecal samples.

The first goal of this study was to investigate the possibility to find filarial infections from DNA extracted from faecal samples of six species of NHPs living in forest regions of Cameroon and Gabon: the gorilla (*Gorilla gorilla gorilla*), the chimpanzee (*Pan troglodyte troglodytes*), the mandrill (*Mandrillus sphinx*), the collared mangabey (*Cercocebus torquatus torquatus*, also known as red-capped mangabey), the agile mangabey (*Cercocebus agilis*) and the greater spot-nosed monkey (*Cercopithecus nictitans*). Secondly, we performed phylogenetic analysis of the filarial DNA fragments found in the faeces of these NHPs, as well as filarial DNA fragments identified in humans to characterise and compare the species. For these purposes, PCRs were carried out by targeting DNA fragments of mitochondrial (*12S* ribosomal DNA and cytochrome *c* oxidase subunit 1 (*cox*1) genes, followed by Sanger sequencing to discriminate parasite species. Lastly, and consequent to the successful amplification of filarial mitochondrial DNA in faeces, we investigated whether DNA presence was associated with co-infections with strongylid nematodes including hookworms [12, 13]. These parasites can cause significant blood loss when adults attach themselves to the intestinal mucosa and while feeding [14]. Despite the absence of impact of strongylid infections on the detection of *Plasmodium* spp. in faeces of western lowland gorillas and eastern chimpanzees [15], their presence and resulting bleeding in the intestine would be a plausible explanation for the detection of filarial DNA in faeces. Therefore, we ran nested PCRs targeting the internal transcribed spacer 2 (ITS2) to look for the presence of infections with *Oesophagostomum* sp. and *Necator* sp. to infer whether there was a positive association between these parasites and the detection of filarial DNA in faeces.

## Methods

### Study sites and faecal collection methods

**Fig. 1:**
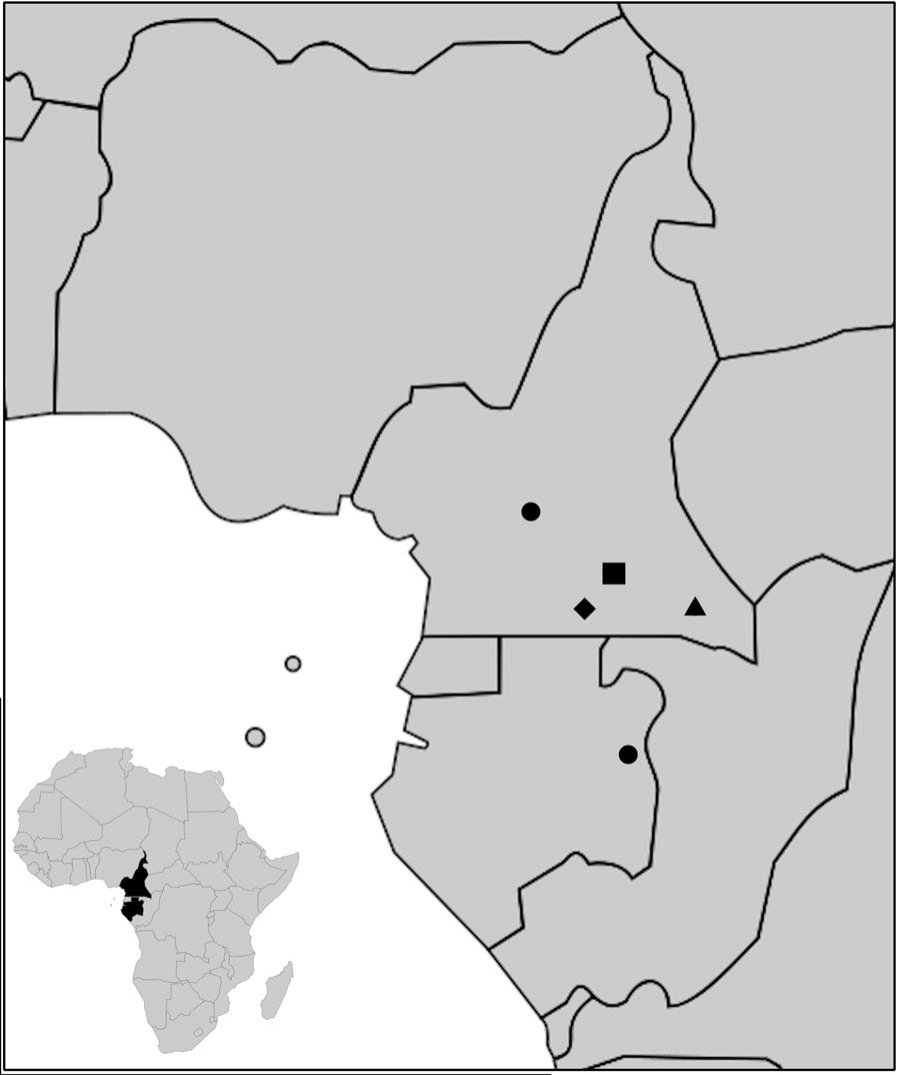
Faecal samples collection sites in Cameroon and in Gabon. In Cameroon: Okola (dot), Djoum (diamond), Somalomo (square), Mambelé/Lobeké National Park (triangle); in Gabon: Matakamangoye (dot)

A total of 315 faecal samples from wild-living, non-habituated NHPs were analysed. These samples were collected non-invasively around NHP nests, feeding places, or on traces at four study sites: the forests surrounding the villages of Djoum (DJ) (2° 40′ 00″ N, 12° 40′ 00″ E), Mambélé-Lobeké National Park (MB/LB) (2° 25′ 00″ N, 15° 24′ 00″ E) and Somalomo (SL) (3° 23′ 00″ N, 12° 44′ 00″ E) in Cameroon (collections between 2008 and 2010), and in remote primary forests and secondary forests surrounding the village of Matakamangoye II (0° 6′ 30″ S, 13° 41′ 57″ E, Gabon (collections between 2009 and 2013) (Fig. 1). These samples were characterized and analysed in previous studies to determine the prevalence and genetic diversity of simian immunodeficiency viruses (SIV) [16, 17]. Faecal samples were identified to be of likely chimpanzee, gorilla or Cercopithecidae monkey origin by experienced trackers and/or by the researchers. A 15–20 g sample was then placed into a 50 ml tube and mixed with an equal amount of RNAlater® (Ambion, Austin, TX, USA). The date, time, and location (longitude and latitude provided by global positioning system, GPS) of sample collection were recorded along with the collector’s name. Faecal samples were generally kept at ambient temperature for no longer than 2 weeks and subsequently stored at − 20 °C once back in Yaoundé, Cameroon, or Franceville, Gabon. Samples were shipped to Montpellier, France, at ambient temperature, and then stored at − 80 °C upon reception. All samples were transported to France in full compliance with export and import regulations.

### DNA extraction from NHP faecal samples

Faecal DNA was extracted using the QIAamp Stool DNA Mini Kit (Qiagen, Valencia, CA, USA) following the manufacturer’s instructions. Briefly, 1.5 ml of faecal RNAlater® mixture was re-suspended in stool lysis buffer and clarified by centrifugation. The supernatants were treated with an InhibitEx tablet, subjected to proteinase K digestion, and passed through a DNA binding column. Bound DNA was eluted in 100 μl elution buffer. To investigate the presence of gastro-intestinal parasites, a modified DNA extraction protocol was adopted, after the clarification. This step consisted of an additional homogenization in tubes containing silica beads of different size (lysis matrix E) in a FastPrep-24 mill (MP Biomedical, Eschwege, Germany) and an overnight incubation at 56 °C to better expose the parasite DNA in eggs.

### Host species confirmation and identification of individual genotypes by microsatellite analyses

Each NHP species and the number of individual faecal samples collected were determined in previous studies, as mentioned before. DNA was extracted from all faecal samples collected in the field and host species were confirmed by PCR amplification and sequence analysis of a 460–500-bp mitochondrial DNA fragment spanning the *12S* rDNA region, using methods described in previous studies [18]. To determine the number of individuals sampled, the faeces collected from gorillas and chimpanzees were subjected to microsatellite analyses, as previously described [17, 19, 20]. Samples were genotyped at seven loci in two multiplex PCRs (amplifying D18s536, D4s243, D10s676, and D9s922 or D2s1326, D2s1333 and D4s1627), and for sex determination, a region of the amelogenin gene that contains a deletion in the X but not the Y chromosome was amplified using a *Taq* DNA polymerase core kit (MP Biomedical, Irvine, CA, USA) with 2 to 10 µl fecal DNA. Mandrills, collared and agile mangabeys and greater spot-nosed monkeys were subjected to mtDNA analyses but not to microsatellite analyses; to ascertain that we were not going to analyze more than one sample belonging to the same individual, we selected the samples among a wide range of samples previously collected and we applied discriminative criteria choices, such as exclusion of primates’ territory ranges overlap and GPS coordinates of the collection locations.

### DNA extraction from human dried blood spots (DBS)

DBS collected from 20 human subjects with known *M. perstans* infection and living in the Okola health district (Cameroon) were shipped to Montpellier to be used as references for phylogenetic analyses [3]. DNA extraction from DBS was performed using the NucliSENS® kit (bioMérieux, Craponne, France) according to the manufacturer’s instructions, as previously described [21]. DNA was eluted in 60 µl buffer. The PCR reactions on the *M. perstans* mf-positive human blood samples were carried on once all other samples had been tested.

### PCR screening of filarial nematodes

A DNA barcoding approach based on either the *cox*1 marker or the *12S* rDNA marker was used to discriminate between filaroid species [2, 8, 22–24]. PCR screening of filarial DNA was based on partial sequences of two mitochondrial genes: *12S* rDNA (approximately 450 bp) and cytochrome *c* oxidase subunit 1 (*cox*1) (*c.*600 bp). Amplification of the *12S* rDNA and *cox*1 fragments was conducted according to Casiraghi et al. [25]. Primers and PCR programmes are shown in Table 1. PCRs were processed in a final volume of 50 μl. The amplification of the target fragments was carried out by nested PCR. Bovine serum albumin (BSA) was added at the final concentration of 0.2 μg/ml to improve amplification success. All PCR reactions were performed using the Expand Long Template PCR system (Roche Diagnostics, Indianapolis, IN, USA), according to the manufacturer’s instructions. Each PCR reaction was carried out on a Bio-Rad T100™ thermal cycler (Hercules, CA, USA) in the presence of a negative control. NHP samples were tested first in a human filaria-free environment. Once all NHP samples were tested, we proceeded with the *M. perstans* mf-positive human blood samples. Amplified fragments were visualized by horizontal electrophoresis on 1% agarose under a UV light. The PCR products of interest were subsequently purified and extracted using the GeneClean® Turbo Kit (Qbiogene Inc., Carslbad, CA, USA). Sequencing reactions were performed with the ABI PRISM® BigDye™ Terminators Kit (Applied Biosystems, Foster City, CA, USA). DNA was then precipitated and sequenced according to the Sanger method (3500 Genetic Analyzer; Applied Biosystems, Foster City, CA, USA).

**Table 1:** Primers and PCR programs

Target organism	Gene	Primer name	Sequence (5′–3′)	Product size (bp)	Thermal profile*						
					Step 1		Step 2		Step3		N
					T	D	T	D	T	D	
Filariae	*12S* rDNA	12SdegF2/	ATTACYTATTYTTAGTTTA	~ 600	94	30	45	30	68	45	45^b^
		12SnemR2^a^	CTACCATACTACAACTTACGC								
		12SF/	GTTCCAGAATAATCGGCTA	~ 450	94	30	54	30	68	30	35
		12SdegR	ATTGACGGATGRTTTGTACC								
	*cox*1	FCo1extdF1	TATAATTCTGTTYTDACTA	~ 970	94	30	45	30	68	60	45^b^
		FCo1extdR1^a^	ATGAAAATGAGCYACWACATAA								
		COIintF/	TGATTGGTGGTTTTGGTAA	~ 650	94	30	54	30	68	45	35
		COIintR	ATAAGTACGAGTATCAATATC								
Hook/ Nodular worms	ITS2	NC1	ACGTCTGGTTCAGGGTTGTT	~ 900	94	30	50	30	72	45	45
		NC2	TTAGTTTCTTTTCCTCCGCT								
		OesophITS2	TGTRACACTGTTTGTCGAAC	250–300	94	30	55	30	72	30	35
		NC2	TTAGTTTCTTTTCCTCCGCT								

^a^ITS2 PCRs start with 95 °C for 15 min and finish by 72 °C for 10 min; *12S* rDNA and *cox*1 PCRs start with 94 °C for 3 min and finish with 68 °C for 10 min

^b^35× for DBS and 45× for stool samples

*Abbreviations*: Step 1, denaturation; Step 2, annealing; Step 3, elongation; T, temperature (°C); D, duration (s); N, number of cycles

### PCR screening of *Oesophagostomum* sp. and *Necator* sp

We ran a semi-nested PCR targeting fragments of the ITS2 maker to determine the presence of *Oesophagostomum* sp. and *Necator* sp. The external PCR was performed using primers NC1 and NC2. Subsequently, a semi-nested internal PCR generating 260 to 350 bp amplicons was performed with primers NC2 and OesophITS2-21 [26–28]. Primers and PCR programs are shown in Table 1. The PCR reaction was performed in a 50 μl reaction volume containing 10 μl and 5 μl of template DNA for primary and semi-nested PCR, respectively, 10 pM of each primer, 25 μl HotStarTaq Master Mix (Qiagen, Courtaboeuf, France), providing a final concentration of 1.5 mM MgCl_2_ and 200 μM of each dNTP. For the external PCR, bovine serum albumin (Sigma-Aldrich, St. Louis, MO, USA) was added at the final concentration of 0.2 μg/ml to improve amplification success. The reactions were performed on a Bio-Rad T100™ thermal cycler (Bio-Rad). Primer details and PCR conditions are listed in Additional file 1: Table S1. The amplicons were electrophoresed on 1% agarose gels stained with ethidium bromide. After purification (on a 2% agarose gel) using the GeneClean® Turbo Kit (Qbiogene), the PCR products were sequenced with the primers of the second step on an automated sequencer (3500 Genetic Analyzer; Applied Biosystems) and the resulting sequences analysed with SeqMan DNASTAR software (Lasergene; DNASTAR, Inc., Madison, WI, USA).

### Molecular identification and phylogenetic analysis

The sequences obtained were assembled, aligned and corrected manually using the SeqMan DNASTAR software (Lasergene, DNASTAR Inc., Madison, WI) and then compared against reference sequences deposited in GenBank using the Nucleotide Basic Local Alignment Search Tool (BLASTn) [29]. Sequences generated during the current study and previously published sequences from draft or complete genomes were aligned using MUSCLE 16 in MEGA 6.06, with minor manual corrections [30]. Maximum Likelihood (ML) was used to infer *cox*1 and *12S* rDNA phylogenetic trees, and were executed with 1000 bootstrap replicates in MEGA 6.06. A partitioned model was implemented to estimate evolution parameters separately for each gene. Using the corrected version of the Akaike information criterion (AIC), JModelTest analysis [31] was performed to establish the evolutionary model best adapted to the sequences alignment for each individual gene and for the concatenation of the two genes. The general time-reversible plus invariant sites plus gamma distributed model (GTR+I+Γ) offered the best fit for *cox*1 and *12S* rRNA. To root the trees, *Filaria latala* (Spirurida: Filariidae) was included as the outgroup. Molecular species delimitation was evaluated using distance-based methods. Between-groups mean distance was calculated using MEGA 6.06.

### Statistical analysis

A two-tailed Fisher’s exact test was used to test for deviations from the null hypothesis, which assumed that there was no association between infection with intestinal parasites and detection of filarial DNA. The tests were applied to each of the different species of NHP separately, as well as to the pooled data across all NHP species.

## Results

### PCR-positive results and filariae homology in *cox*1 and *12S* rDNA fragments from six species of non-human primates

The 315 samples of NHP faeces included 65 samples from gorillas, 12 from mandrills, 222 from chimpanzees, 9 from red-capped mangabeys, 3 from agile mangabeys and 4 from greater spot-nosed monkeys. PCRs targeting the *12S* rDNA and *cox*1 mitochondrial genes of filariae were run for all samples selected. *12S* rDNA and *cox*1 sequences were also obtained from 20 human patients and 7 of them were added for comparisons to the phylogenetic trees. Among the 315 samples analysed, 121 were positive by PCR (38%): 31 samples were positive for *12S* rDNA only, 59 samples were positive for *cox*1 only, and 31 for both gene fragments (Table 2). The sequences obtained showed > 90% homology with reference filarial sequences available on GenBank (BLASTn). For the gorillas, sequences from 30 out of 65 samples (46%), for the mandrills, 2 sequences out of 12 (17%), for the chimpanzees 85 out of 222 (38%), for the *Cercocebus* only 1 (agile mangabey) out of 12 (8%), and for the greater spot-nosed monkeys 3 out of 4 (75%) showed high similarities (> 90% homology) within the family Onchocercidae.

**Table 2:** PCR and BLASTn summary results according to NHP species and location

Country	Locality	Host species	*n*	Positive *12S* rDNA	Positive *cox*1	Positive *12S* rDNA and *cox*1	Positive individuals
Cameroon	Djoum	Chimpanzee	0	0	0	0	0
		Gorilla	0	0	0	0	0
		Mandrill	12	2	0	0	2
		Red-capped Mangabey	7	0	0	0	0
		Greater spot-nosed monkey	1	0	0	0	0
	Somalomo	Chimpanzee	23	4	9	2	11
		Gorilla	38	12	13	7	18
		Mandrill	0	0	0	0	0
		Red-capped Mangabey	2	0	0	0	0
		Greater spot-nosed monkey	2	0	2	0	2
	Mambelé/ Lobeke NP	Chimpanzee	59	26	20	15	31
		Gorilla	27	4	8	0	12
		Mandrill	0	0	0	0	0
		Agile Mangabey	3	1	1	1	1
		Greater spot-nosed monkey	1	1	1	1	1
Gabon	Matakamangoye	Chimpanzee	140	12	36	5	43
Total			315	62	90	31	121

*Abbreviation*: n, individuals tested

### Phylogenetic analyses of *cox*1 and *12S* rDNA gene fragments

Among these 121 samples, 39 out of 62 sequences were exploitable for phylogenetic analyses for the *12S* rDNA gene (corresponding to 63% of the sequences obtained) and 70 out of 90 for the *cox*1 gene (corresponding to 78% of the sequences obtained) (Table 3). The remaining sequences were not included in the alignments because they were of low quality (less than 250 bp) or displayed background noise with too many unresolved degenerated nucleotides.

**Table 3:** Summary results of positive individuals in phylogenetic trees according to NHP species and location

Country	Locality	Host species	*n*	*12S* rDNA phylogeny	*cox*1 phylogeny	*12S* rDNA and *cox*1 phylogeny	Positive individuals
Cameroon	Djoum	Chimpanzee	0	0	0	0	0
		Gorilla	0	0	0	0	0
		Mandrill	12	1	0	0	1
		Red-capped Mangabey	7	1	0	0	1
		Greater spot-nosed monkey	1	0	0	0	0
	Somalomo	Chimpanzee	23	4	7	1	10
		Gorilla	38	11	15	2	24
		Mandrill	0	0	0	0	0
		Red-capped Mangabey	2	0	0	0	0
		Greater spot-nosed monkey	2	0	2	0	2
	Mambelé/Lobeke NP	Chimpanzee	59	11	14	2	23
		Gorilla	27	1	9	0	10
		Mandrill	0	0	0	0	0
		Agile Mangabey	3	1	1	1	1
		Greater spot-nosed monkey	1	0	1	0	1
Gabon	Matakamangoye	Chimpanzee	140	9	21	0	30
Total			315	39	70	6	103

*Abbreviation*: n, individuals tested

**Fig. 2:**
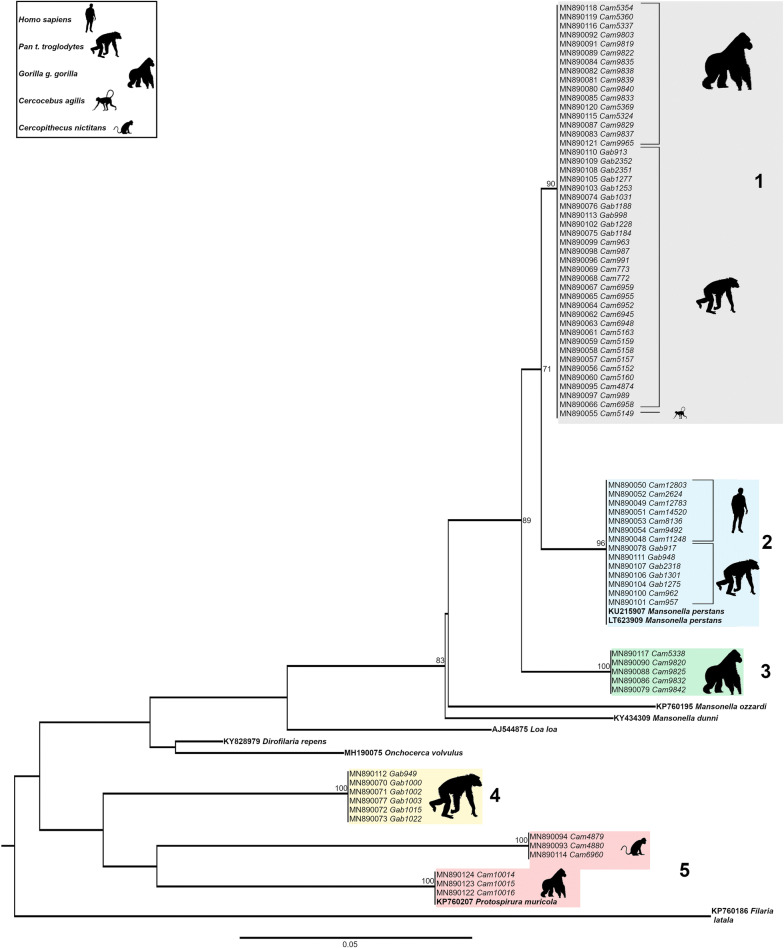
Phylogenetic tree based on cytochrome *c* oxidase subunit 1 (*cox*1) gene sequences from 85 onchocercid specimens. The total length of the alignment is 474 bp. *Filaria latala* was included as the outgroup. The topology was inferred using Maximum Likelihood. Nodes are associated with bootstrap values based on 1000 replicates. Bootstrap support values < 70 are not shown. The scale-bar indicates the number of nucleotide substitutions. Reference specimens are in bold

Phylogenetic analyses of the 70 *cox*1 gene fragments depicted a tree composed of 5 monophyletic groups (Fig. 2). At the bottom (group 5), a group of three gorilla samples clustered together with *Protospirura muricula* (Nematoda: Spiruridae, a parasite of murid rodents) and 3 other samples from *Cercopithecus nictitans* branched out of the same node. The group just above, represented exclusively by chimpanzee faecal samples from Gabon (group 4) was not very highly supported, its robustness being < 70. Therefore, it remains difficult to associate these sequences to a given species. Then, three groups (1, 2 and 3) clustered together within the genus *Mansonella* with a bootstrap support > 70%. They all belong to the clade ONC5 as described in Lefoulon et al. [2] (Additional file 2: Figure S1). Parasite sequences from five chimpanzees from Matakamangoye (Gabon) and two chimpanzees from Somalomo (Cameroon) grouped together with *M. perstans* infecting humans from the Okola region (Cameroon) and with two *M. perstans* reference sequences infecting humans, retrieved from the GenBank (group 2). Sample homology was > 98%. Groups 1 and 3 were composed of nematodes of the genus *Mansonella* infecting gorillas, chimpanzees and mangabeys from different regions (Matakamangoye-Gabon, Somalomo and Mambele/Lobeke National Park-Cameroon), indicating the absence of phylogeographic clusters. In *cox*1, group 1 and group 2 sequences were genetically relatively closer than groups 2 and 3, with a mean interspecific divergence of 4.2%, and 6.8% respectively. Identification of filarial species using *cox*1 as a molecular marker was accurate, as previously indicated for other filarial species [2, 8, 22]. The *cox*1 divergence threshold value to discriminate between onchocercid species was previously established at 4.8% [22]. However, depending on the dataset and the number of analysed sequences, intraspecific distances between most of the studied onchocercid species were lower than 2% and interspecific distances were higher than 4.5%.

**Fig. 3:**
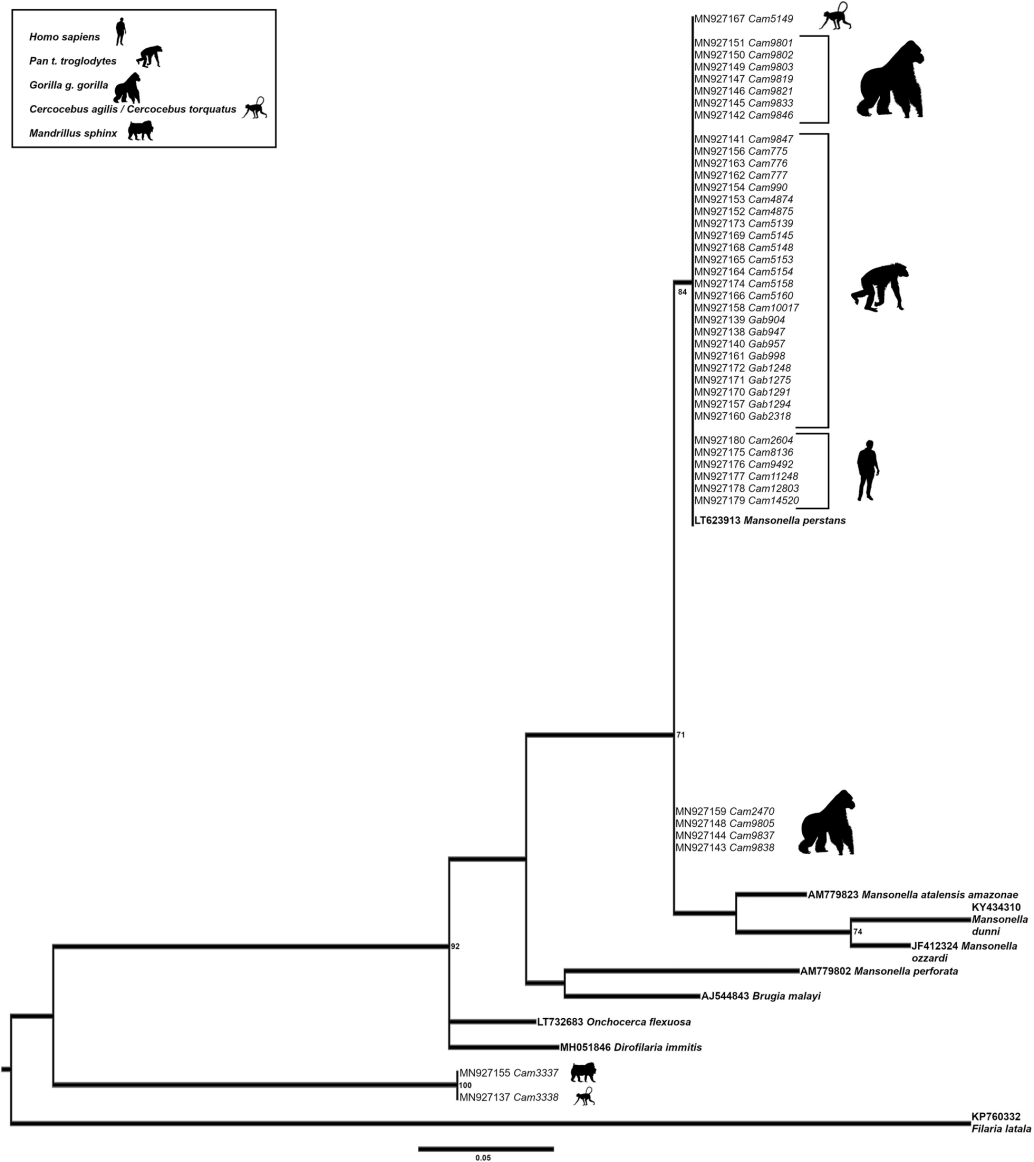
Phylogenetic tree based on *12S* rDNA gene sequences from 52 onchocercid specimens. The total length of the alignment is 266 bp. *Filaria latala* was included as the outgroup. The topology was inferred using Maximum Likelihood. Nodes are associated with bootstrap values based on 1000 replicates. Bootstrap support values < 70 are not shown. The scale-bar indicates the number of nucleotide substitutions. Reference specimens are in bold

Phylogenetic analyses of the *12S* rDNA gene fragment showed less discriminating power compared to the analyses of the *cox*1 fragment and fewer sequences were available for analysis (Fig. 3, Table 3) as observed for the analysis of the genus *Onchocerca* [8], therefore precise species identification remains unresolved. However, 6 samples (CAM_5149, CAM_9803, CAM_9838, CAM_5158, CAM_5160 and CAM_4874) within group 1 in the *cox*1 phylogenetic tree, have been successfully amplified for the *12S* rDNA fragment. Five out of these 6 samples amplified in both genes, clustered together with *M. perstans* samples infecting humans, although with a less robust bootstrap support; only sample CAM_9838, from a gorilla, branched outside this *M. perstans* group in the *12S* phylogenetic tree. Finally, 2 samples from Djoum in Cameroon (CAM_3337 from a mandrill and CAM_3338 from a mangabey) branched together but outside of the known filariae and *Protospirura muricola* DNA references.

### Filarial DNA detection in faeces and co-infections with gastro-intestinal nematodes

Regarding the intestinal nematodes, 106 of the 121 NHPs found to be positive for filariae (87.6%) were also infected either with *Oesophagostomum* sp., or *Necator* sp., or both. In contrast, among the 194 samples in which we could not detect any filariae signals, 151 (77.8%) were infected with *Oesophagostomum* sp., or *Necator* sp. or both. There was no statistically significant association between the presence of infection with these intestinal nematodes and detection of filarial DNA when all NHP species were analysed together or when *Cercopithecus* sp. and *Cercocebus* sp. monkeys, mandrills or chimpanzees were analysed separately. However, for the gorillas, we detected significantly more filarial infections when the animals were also infected with gastro-intestinal worms (two-tailed probability = 0.0125; Tables 2, 4, 5).

**Table 4:** Association between filariae detection (*12S* rDNA and *cox*1) and gastro-intestinal worm infection (ITS2)

NHP species	No. of samples tested	*12S*/*cox*1 pos/ITS2neg	*12S*/*cox*1 pos/ITS2pos	*12S*/*cox*1 neg/ITS2neg	1*2S*/*cox*1 neg/ITS2pos
*Cercopithecus nictitans*	4	0	3	1	0
*Cercobebus torquatus/agilis*	12	0	1	5	6
*Pan troglodytes*	222	15	70	26	111
*Gorilla gorilla*	65	0	30	7	28
*Mandrillus sphinx*	12	0	2	4	6
Total	315	15	106	43	151

*Abbreviations*: neg, negative; pos, positive

**Table 5:** Fisher exact test contingency table and two-tailed probability

Species	A	C	Two-tailed probability
	B	D	
*Cercopithecus* sp.*/Cercobebus* sp.	0	4	0.233
	6	6	
*Pan troglodytes*	15	70	0.860
	26	111	
*Gorilla gorilla*	0	30	0.0125
	7	28	
*Mandrillus sphinx*	0	2	0.515
	4	6	
TOTAL	15	106	0.036
	43	151	

*Abbreviations*: A, positive for filariae and negative for GI worms; B, negative for filariae and negative for GI worms; C, positive for filariae and positive for GI worms; D, negative for filariae and positive for GI worms

## Discussion

To the best of our knowledge, this is the first study showing that filarial DNA can be detected in the faeces of NHPs using a molecular approach. Among the 315 samples analysed, 121 (38.4%) showed > 90% homology with Onchocercidae reference sequences. The true infection rates are likely to be even higher, because filariae detection in faecal samples is probably less sensitive than detection in blood. Phylogenetic analyses on *cox*1 sequences obtained from five chimpanzee DNA faecal samples from Gabon and two from Cameroon show that these samples cluster together with *Mansonella perstans* with high bootstrap support. Most of the remaining sequences clustered together within the genus *Mansonella*, but the species could not be resolved. It has to be noted that most of the studies on filariae infecting NHPs are limited to morphological descriptions and taxonomic analyses at the molecular level are lacking. In wild-living NHPs, adult filariae have been recovered after extraction from lymphatic vessels during necropsies, but these findings remain rare [32]. Because of their localization in host tissues, *Mansonella* adult stages are difficult to collect and the diversity of this genus is far from being well investigated in animals. Up to now, seven species of *Mansonella* (*M. perstans*, *M. streptocerca*, *M. leopoldi*, *M. lopeensis*, *M. rodhaini*, *M. gorillae* and *M. vanhoofi*) have been described in anthropoids and humans from Africa, based on morphological characteristics only. They all belong to the subgenus *Esslingeria* [1]. Given the difficulty to recover adult filariae in NHPs, indirect approaches targeting microfilarial DNA could be helpful for their identification [33, 34]. In this study, *cox*1 phylogenetic analyses were more informative than *12S* rDNA, with sequence fragments of 480 and 280 bp, respectively. In this study, we found seven chimpanzee faecal samples infected with *M. perstans*, a filaria found in humans, while 29 samples of chimpanzees and 21 of gorillas were infected with a *Mansonella* sp. forming a separate cluster in the *cox*1 phylogenetic tree. Using also a DNA barcoding approach based on the ITS marker, a putative new *Mansonella* species/subspecies was detected exclusively in febrile children from Gabon [35]. However, by adopting a Sanger sequencing approach, the identification of co-infections with different *Mansonella* species cannot be resolved in a given individual. In a past morphological study, four *Mansonella* species were identified in a single male western lowland gorilla (*G. gorilla gorilla*) found dead in the Lopé Reserve, Gabon [32]. In this animal, female worms of two species of *Mansonella* (*M. lopeensis* and *Mansonella* sp.) were recovered from the deep tissues of the wounded thigh, a single *M. leopoldi* mf was found in the blood vessels of the liver, and a male of *Mansonella* sp. was situated in an afferent lymphatic vessel of an axillary lymph node. Future efforts using next-generation sequencing (NGS) could lower the threshold of detection and improve the sensitivity for detecting low-frequency variants. To date, 14 filarial genomes have been assembled by an NGS approach. A phylogenetic analysis of 41 nematode genomes has been performed, with a consensus tree including 11 out of 14 filarial assemblies. However, no *Mansonella* sp. assemblies are available yet [36]. Phylogenetic trees have also been constructed from filarial parasite mitogenome sequences [37, 38], comforting the clades described by Lefoulon et al. [2] and the presence of *Mansonella* sp. in ONC5. The NGS method would also be more suitable to discriminate between a possible multitude of *Mansonella* spp. parasites and mixed infections with multiple filarial species. Deep sequence analysis represents a potential tool to detect occult infections, as reported in a study where samples classified by traditional methods as *M. perstans* mono-infections were found, through rtPCR and deep sequencing, to also have occult *M. ozzardi* infections [38].

*Mansonella perstans* adult filariae are mainly found in in the serous cavities, the visceral adipose tissue or the subcutaneous tissue. However, the microfilariae thrive in the blood and the lymphatic system [39], therefore their detection (or the detection of filarial DNA fragments) in faecal samples, seems surprising. However, a few reports have shown that the adult parasites are usually found in the mesenteries and connective tissues of abdominal organs of humans, gorillas and chimpanzees [34, 40–43]. In the present study, the detection of parasite DNA in the faecal samples seem not to be associated with the presence of gastrointestinal worms such as *Oesophagostomum* sp. or *Necator* sp., except possibly in the gorilla population analysed. The magnitude of *Necator* sp.-associated pathogenesis and blood loss is highly dependent on the number of adult worms, host immunity and concurrent infections with other nematodes [44]. To properly estimate infection intensity with a particular gastro-intestinal parasite species, invasive collection of adult parasites from the intestines of infected hosts is required, which is indeed impossible in wild endangered animals like apes, with exception of necropsies. Regarding the presence of *Oesophagostomum*, these infections are usually not associated with intestinal bleeding, although a study reports rectal bleeding due to *Oesophagostomum brumpti* in a human individual [45] and another study reported intestinal perforation in severe cases of oesophagostomiasis in gorillas [46]. In general, data about clinical manifestations and pathogenesis of any strongylid infections in wild NHPs are scarce [47]. Therefore, it remains difficult to draw any conclusion about the association of gastrointestinal worms and the detection of filarial DNA in faeces from gorillas. Studies investigating phytochemical and mechanical properties of plants consumed by NHPs suggest the existence of abrasive properties of certain foliage [47], which could favour the bleeding of the intestine wall and the release of mf in this body compartment [48]. Lastly, we cannot exclude that the successful detection of filarial DNA in faecal samples may be explained by the presence of cell-free DNA (cfDNA). CfDNA comprises fragments of DNA found extracellularly in the blood circulation but also in different body fluids and tissues. Despite different hypotheses having been presented, the precise origin of cfDNA and how it is distributed remains unclear [49, 50].

Finally, we detected the spirurid nematode *Protospirura muricola* in faecal samples from gorillas and greater spot-nosed monkeys. Eggs and fragments of the posterior body of an adult male of *P. muricola* have been found in chimpanzees’ faecal samples [51]. *Protospirura muricola* is a relatively frequent parasite of rodents in Africa and Asia [52–55]. Adults are found in the stomach of the definitive hosts [56]. Dermapterans and most probably also scarabeid beetles are intermediate hosts in the life-cycle of the nematode [57]. Therefore, we cannot exclude with certainty that these faecal samples, laying on the ground before collection, could have been contaminated by *P. muricola* infecting dung beetles or by faecal samples from rodents (the gorilla samples were collected along a road about 500 m from a small house settlement (2° 26′ 15.828″ N, 15° 26′ 19.5″ E) and the *C. nictitans* sample in a cocoa plantation near the village of Mambele (2° 23′ 54.492″ N, 15° 30′ 43.524″ E). However, this parasite has been previously described also in wild NHPs from Nigeria [53] and the Congo [58], in re-introduced chimpanzees in Tanzania [51] and in captive NHPs from south America [59, 60]. Though *P. muricola* in rodents seems to be a relatively non-pathogenic parasite, it causes severe, sometimes fatal, disease in captive primates. However, the pathogenicity of the nematode in wild chimpanzees or gorillas remains unknown and it has not been reported in humans.

## Conclusions

To the best of our knowledge, this study shows for the first time that filarial infections can be diagnosed from DNA extracted from faecal material in several NHP species. These preliminary results open up perspectives on the detection of Onchocercidae in samples other than blood and dermal tissues. Such non-invasive sampling, combined with molecular genetic analysis, could allow for investigations of these pathogens in wild animal populations that are difficult to access. Moreover, our results raise questions about the diversity and abundance of these parasites in wildlife and their possible role as sylvatic reservoirs. Similar to other pathogens, which have crossed the species barrier, zoonotic filariae transmission seems possible and could potentially increase [61], especially considering human encroachment into previously pristine territories and the growing proximity between humans and wild animals. Future studies should focus on improving the sensitivity of the tests in both human and NHPs, and improve and extend the molecular information to resolve or support taxonomy classification based on morphological descriptions. Finally, this non-invasive approach should be further tested as a diagnostic alternative in the human population, especially in situations where people are reluctant to undergo blood analysis or where blood tests would require people to engage in long journeys from remote villages to diagnostic centres. Health operators would collect faecal samples at domicile and test a single sample for multiple infections back in a centralized laboratory.

## Supplementary information

**Additional file 1: Table S1.** Accession number (GenBank) list of new filarial species sequences and reference sequences for phylogenetic analyses (*cox*1 and *12S* rDNA).

**Additional file 2: Figure S1.** Phylogeny of Onchocercidae based on *cox*1 sequences using Maximum Likelihood inference. The total length of the dataset is 475 bp. A total of 86 onchocercid specimens (48 sequences from this study with 38 sequences from 38 species from Lefoulon et al. [2]) were analysed. *Filaria latala* was used as the outgroup. The topology was inferred using Maximum Likelihood, under the general time reversible model, including invariant sites and gamma distribution (GTR+I+Γ). Nodes are associated with bootstrap values based on 1000 replicates. The onchocercid subfamilies are indicated by color: blue for Onchocercinae, dark green for Dirofilariinae, purple for Splendidofilariinae, yellow for Waltonellinae. Although the *cox*1 gene is not informative enough for deeper phylogenies it is for species level phylogenies and onchocercid clades described in Lefoulon et al. [2] are identifiable, especially ONC5 to which belong the new sequences.

## Data Availability

All data generated or analysed during this study are included in this article and its additional files. We deposited all newly generated sequences from this study in the GenBank database under the accession numbers: MN890048-MN890124 (*cox*1) and MN927137-MN927180 (*12S* rDNA) (Additional file 1: Table S1). The datasets on *Oesophagostomum* spp. and *Necator* spp. used and/or analysed during the present study are available from the corresponding author on reasonable request.

## Abbreviations

AIC: Akaike information criterion
BLASTn: Nucleotide Basic Local Alignment Search Tool
BSA: Bovine serum albumin
cfDNA: cell-free DNA
*cox*1: cytochrome *c* oxidase subunit 1
GTR + I + Γ: general time-reversible plus invariant sites plus gamma distributed model
ITS2: internal transcribed spacer 2
LAMP: loop-mediated isothermal amplification
ML: Maximum Likelihood
mf: microfilariae
NGS: next-generation sequencing
NHP: non-human primates
(rt)PCR: (real time) polymerase chain reaction
SIV: simian immunodeficiency virus
