# Supplementary material for: Detection of DNA of filariae closely related to Mansonella perstans in faecal samples from wild non-human primates from Cameroon and Gabon
Source: Parasit Vectors. 2020 Jun 16;13:313. doi: 10.1186/s13071-020-04184-1 (PMC7298833; doi:10.1186/s13071-020-04184-1)
Supplement: Supplementary file 1 — Additional file 1: Table S1. Accession number (GenBank) list of new filarial species sequences and reference sequences for phylogenetic analyses (cox1 and 12S rDNA). [file 13071_2020_4184_MOESM1_ESM.pdf]

Additional file 1: Table S1

**Host species**	**Sequence ID**	**#Accession (*cox*1)**
H.sapiens	*Cam11248*	MN890048
H.sapiens	*Cam12783*	MN890049
H.sapiens	*Cam12803*	MN890050
H.sapiens	*Cam14520*	MN890051
H.sapiens	*Cam2624*	MN890052
H.sapiens	*Cam8136*	MN890053
H.sapiens	*Cam9492*	MN890054
C.agilis	*Cam5149*	MN890055
P.t.troglodytes	*Cam5152*	MN890056
P.t.troglodytes	*Cam5157*	MN890057
P.t.troglodytes	*Cam5158*	MN890058
P.t.troglodytes	*Cam5159*	MN890059
P.t.troglodytes	*Cam5160*	MN890060
P.t.troglodytes	*Cam5163*	MN890061
P.t.troglodytes	*Cam6945*	MN890062
P.t.troglodytes	*Cam6948*	MN890063
P.t.troglodytes	*Cam6952*	MN890064
P.t.troglodytes	*Cam6955*	MN890065
P.t.troglodytes	*Cam6958*	MN890066
P.t.troglodytes	*Cam6959*	MN890067
P.t.troglodytes	*Cam772*	MN890068
P.t.troglodytes	*Cam773*	MN890069
P.t.troglodytes	*Gab1000*	MN890070
P.t.troglodytes	*Gab1002*	MN890071
P.t.troglodytes	*Gab1015*	MN890072
P.t.troglodytes	*Gab1022*	MN890073
P.t.troglodytes	*Gab1031*	MN890074
P.t.troglodytes	*Gab1184*	MN890075
P.t.troglodytes	*Gab1188*	MN890076
P.t.troglodytes	*Gab1003*	MN890077
P.t.troglodytes	*Gab917*	MN890078
G.g.gorilla	*Cam9842*	MN890079
G.g.gorilla	*Cam9840*	MN890080
G.g.gorilla	*Cam9839*	MN890081
G.g.gorilla	*Cam9838*	MN890082
G.g.gorilla	*Cam9837*	MN890083
G.g.gorilla	*Cam9835*	MN890084
G.g.gorilla	*Cam9833*	MN890085
G.g.gorilla	*Cam9832*	MN890086
G.g.gorilla	*Cam9829*	MN890087
G.g.gorilla	*Cam9825*	MN890088
G.g.gorilla	*Cam9822*	MN890089
G.g.gorilla	*Cam9820*	MN890090
G.g.gorilla	*Cam9819*	MN890091
G.g.gorilla	*Cam9803*	MN890092
C. nictitans	*Cam4880*	MN890093

C. nictitans	*Cam4879*	MN890094
P.t.troglodytes	*Cam4874*	MN890095
P.t.troglodytes	*Cam991*	MN890096
P.t.troglodytes	*Cam989*	MN890097
P.t.troglodytes	*Cam987*	MN890098
P.t.troglodytes	*Cam963*	MN890099
P.t.troglodytes	*Cam962*	MN890100
P.t.troglodytes	*Cam957*	MN890101
P.t.troglodytes	*Gab1228*	MN890102
P.t.troglodytes	*Gab1253*	MN890103
P.t.troglodytes	*Gab1275*	MN890104
P.t.troglodytes	*Gab1277*	MN890105
P.t.troglodytes	*Gab1301*	MN890106
P.t.troglodytes	*Gab2318*	MN890107
P.t.troglodytes	*Gab2351*	MN890108
P.t.troglodytes	*Gab2352*	MN890109
P.t.troglodytes	*Gab913*	MN890110
P.t.troglodytes	*Gab948*	MN890111
P.t.troglodytes	*Gab949*	MN890112
P.t.troglodytes	*Gab998*	MN890113
C. nictitans	*Cam6960*	MN890114
G.g.gorilla	*Cam5324*	MN890115
G.g.gorilla	*Cam5337*	MN890116
G.g.gorilla	*Cam5338*	MN890117
G.g.gorilla	*Cam5354*	MN890118
G.g.gorilla	*Cam5360*	MN890119
G.g.gorilla	*Cam5369*	MN890120
G.g.gorilla	*Cam9965*	MN890121
G.g.gorilla	*Cam10016*	MN890122
G.g.gorilla	*Cam10015*	MN890123
G.g.gorilla	*Cam10014*	MN890124
H.sapiens	*Mansonella perstans*	KU215907
H.sapiens	*Mansonella perstans*	LT623909
H.sapiens	*Mansonella ozzardi*	KP760195
Not specified	*Mansonella dunni*	KY434309
Not specified	*Loa loa*	AJ544875
Canis lupus familiaris	*Dirofilaria repens*	KY828979
H.sapiens	*Onchocerca volvulus*	MH190075
Gorilla sp.	*Protospirura muricola*	KP760207
Panthera leo	*Filaria lalata*	KP760186
Agama agam	*Foleyella candezei*	KP760187
Ateles sp.	*Dipetalonema caudispina*	KP760178
Bos taurus	*Onchocerca armillata*	KP760200
B. taurus	*Onchocerca gutturosa*	KP760201
B. taurus	*Onchocerca ochengi*	KP760202
Canis familiaris	*Cercopithifilaria bainae*	KP760175
Capreolus capreolus	*Cercopithifilaria rugosicauda*	KC610815
Cariollia perspicillata	*Litomosoides brasiliensis*	KP760190
Cebus olivaceus	*Dipetalonema gracile*	KP760179
Cervus nippon	*Mansonella perforata*	AM749265

C. nippon	*Onchocerca eberhardi*	AM749268
C. nippon	*Onchocerca skrjabini*	AM749269
Glossophaga soricina	*Litomosoides hamletti*	KP760192
H.sapiens	*Brugia timori*	KP760171
H.sapiens	*Loa loa*	KP760194
Hydrochoerus hydrochaeris	*Cruorifilaria tuberocauda*	KP760176
H. hydrochaeris	*Yatesia hydrochoerus*	KP760210
Lagothrix peoppigi	*Dipetalonema robini*	KP760183
Meriones unguiculatus	*Brugia malayi*	KP760171
M. unguiculatus	*Brugia pahangi*	KP760171
M. unguiculatus	*Litomosoides sigmodontis*	mLs2.0
Naemorhedus crispus	*Loxodontofilaria caprini*	AM749237
Not specified	*Onchocerca volvulus*	AF015193
Oriolus oriolus	*Cardiofilaria pavlovskyi*	KP760174
Pachycactylus turneri	*Madathamugadia hiepei*	JQ888270
Phyllomedusa bicolor	*Ochoterenella sp.3*	KP760197
Podiceps nigricollis	*Pelecitus fulicaeatrae*	KP760206
Rangifer tarandus	*Rumenfilaria andersoni*	JQ888273
Rattus tanezumi	*Breinlia jittapalapongi*	KP760170
Rhinella granulosa	*Ochoterenella sp.1*	KP760198
R. marina	*Ochoterenella sp.2*	KP760199
Saltator similis	*Aproctella alessandroi*	FR823335
Samiris scuireus	*Dipetalonema graciliformis*	KP760182
Sus scrofa	*Onchocerca dewittei japonica*	KP760203
Trachops cirrhosus	*Litomosoides solarii*	KP760193

**Host species**	**Sequence ID**	**#Accession *12S* rDNA**
C.torquatus	*Cam3338*	MN927137
P.t.troglodytes	*Gab947*	MN927138
P.t.troglodytes	*Gab904*	MN927139
P.t.troglodytes	*Gab957*	MN927140
G.g.gorilla	*Cam9847*	MN927141
G.g.gorilla	*Cam9846*	MN927142
G.g.gorilla	*Cam9838*	MN927143
G.g.gorilla	*Cam9837*	MN927144
G.g.gorilla	*Cam9833*	MN927145
G.g.gorilla	*Cam9821*	MN927146
G.g.gorilla	*Cam9819*	MN927147
G.g.gorilla	*Cam9805*	MN927148
G.g.gorilla	*Cam9803*	MN927149
G.g.gorilla	*Cam9802*	MN927150
G.g.gorilla	*Cam9801*	MN927151
P.t.troglodytes	*Cam4875*	MN927152
P.t.troglodytes	*Cam4874*	MN927153
P.t.troglodytes	*Cam990*	MN927154
Mandrillus sphinx	*Cam3337*	MN927155
P.t.troglodytes	*Cam775*	MN927156
P.t.troglodytes	*Gab1294*	MN927157
P.t.troglodytes	*Cam10017*	MN927158
G.g.gorilla	*Cam2470*	MN927159
P.t.troglodytes	*Gab2318*	MN927160
P.t.troglodytes	*Gab998*	MN927161
P.t.troglodytes	*Cam777*	MN927162
P.t.troglodytes	*Cam776*	MN927163
P.t.troglodytes	*Cam5154*	MN927164
P.t.troglodytes	*Cam5153*	MN927165
P.t.troglodytes	*Cam5160*	MN927166
C. agilis	*Cam5149*	MN927167
P.t.troglodytes	*Cam5148*	MN927168
P.t.troglodytes	*Cam5145*	MN927169
P.t.troglodytes	*Gab1291*	MN927170
P.t.troglodytes	*Gab1275*	MN927171
P.t.troglodytes	*Gab1248*	MN927172
P.t.troglodytes	*Cam5139*	MN927173
P.t.troglodytes	*Cam5158*	MN927174
H.sapiens	*Cam8136*	MN927175
H.sapiens	*Cam9492*	MN927176
H.sapiens	*Cam11248*	MN927177
H.sapiens	*Cam12803*	MN927178
H.sapiens	*Cam14520*	MN927179
H.sapiens	*Cam2604*	MN927180
Not specified	*Mansonella perstans*	LT623913

C. olivaceus	*Mansonella_atalensis_amazonae*	AM779823
Not specified	*Mansonella_dunni*	KY434310
H.sapiens	*Mansonella_ozzardi*	JF412324
Cervus nippon	*Mansonella_perforata*	AM779802
Not specified	*Brugia_malayi*	AJ544843
Cervus elaphus	*Onchocerca_flexuosa*	LT732683
Lutra lutra	*Dirofilaria_immitis*	MH051846
Panthera leo	*Filaria_lalata*	KP760332
