# Supplementary figures and images for: Detection of DNA of filariae closely related to Mansonella perstans in faecal samples from wild non-human primates from Cameroon and Gabon
Source: Parasit Vectors. 2020 Jun 16;13:313. doi: 10.1186/s13071-020-04184-1 (PMC7298833; doi:10.1186/s13071-020-04184-1)

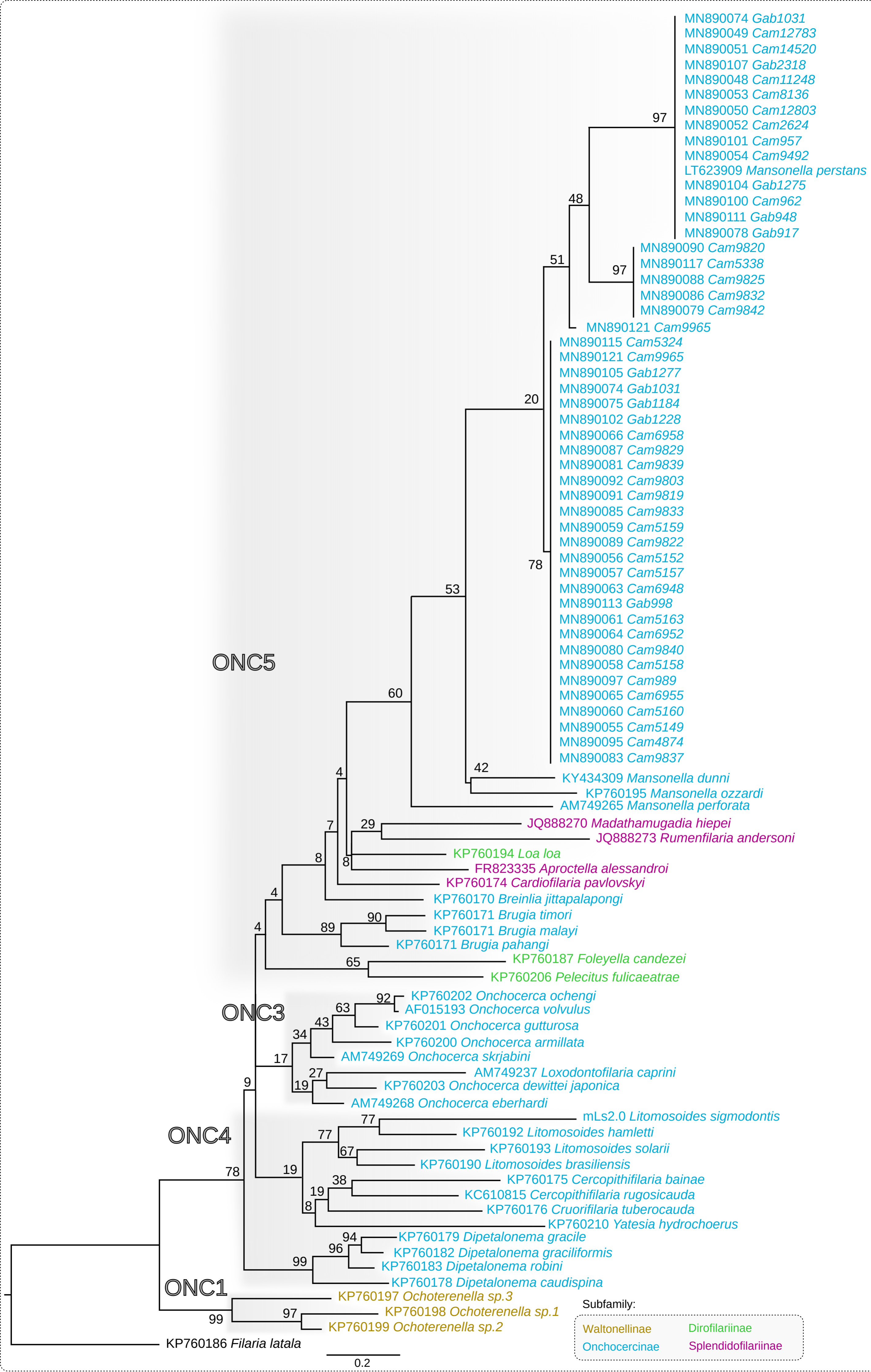

Supplement: Supplementary file 2 — Additional file 2: Figure S1. Phylogeny of Onchocercidae based on cox1 sequences using Maximum Likelihood inference. The total length of the dataset is 475 bp. A total of 86 onchocercid specimens (48 sequences from this study with 38 sequences from 38 species from Lefoulon et al. [2]) were analysed. Filaria latala was used as the outgroup. The topology was inferred using Maximum Likelihood, under the general time reversible model, including invariant sites and gamma distribution (GTR+I+Γ). Nodes are associated with bootstrap values based on 1000 replicates. The onchocercid subfamilies are indicated by color: blue for Onchocercinae, dark green for Dirofilariinae, purple for Splendidofilariinae, yellow for Waltonellinae. Although the cox1 gene is not informative enough for deeper phylogenies it is for species level phylogenies and onchocercid clades described in Lefoulon et al. [2] are identifiable, especially ONC5 to which belong the new sequences. [file 13071_2020_4184_MOESM2_ESM.pdf]
